# Supplementary material for: MetaQTL: a package of new computational methods for the meta-analysis of QTL mapping experiments
Source: BMC Bioinformatics. 2007 Feb 8;8:49. doi: 10.1186/1471-2105-8-49 (PMC1808479; doi:10.1186/1471-2105-8-49)
Supplement: Additional File 7 — MetaQTL Package : jar file and tutorial. This Zip archive contains both the MetaQTL JAR file and the files of the tutorial. [file 1471-2105-8-49-S7.zip › org.inra.metaqtl/doc/index-files/index-18.html]

S-Index


|  |  |  |
| --- | --- | --- |
| **Overview** \| Package \| Class \| Use \| **Tree** \| **Deprecated** \| **Index** \| **Help** | |  |
| **PREV LETTER**   **NEXT LETTER** | **FRAMES**    **NO FRAMES**     **All Classes** |  |


A B C D E F G H I K L M N O P Q R S T U V W X Y Z 

---


## **S**

**s\_mrkIdxL** - Variable in class org.metaqtl.Qtl.QTLProj: The skeleton indice of the left flanking marker. **s\_mrkIdxR** - Variable in class org.metaqtl.Qtl.QTLProj: The skeleton indice of the right flanking marker. **scale(double)** - Method in class org.metaqtl.graph.ChromAxe: Scale the axe in y. **scaleHeight(double)** - Method in class org.metaqtl.graph.ChromAxe: **scaleY(double)** - Method in class org.metaqtl.graph.ChromAxe: **sd** - Variable in class org.metaqtl.MetaQtlResult: The QTL position standard deviations. **sd2ci(double)** - Static method in class org.metaqtl.numrec.NumericalUtilities: **sd\_mode** - Variable in class org.metaqtl.algo.QtlProjAlgorithm: The mode of compuation of the standard deviation of the QTL positions. **sd\_position** - Variable in class org.metaqtl.Qtl: The standard deviation of the QTL position. **sdd** - Variable in class org.metaqtl.MetaChrom: The standard deviation of the position in Morgan. **selfingIndividualInformation(OffspringManager.HaldaneWaddingtonProportion)** - Static method in class org.metaqtl.util.OffspringManager: This routine returns the individual means amount of information for the given generation propertions for a self-fertilization design. **selfingRecurrence(OffspringManager.HaldaneWaddingtonProportion, double)** - Static method in class org.metaqtl.util.OffspringManager: Given the current proportion C,D,E,F and G and the recombination rate r this routine computes the next generation proportions following the recurrence formula of Haldane and Waddington, 1931, Inbreeding and linkage, Genetics 16:357-374 (see section self-fertilization p358). **SEPARATOR** - Static variable in class org.metaqtl.factory.MetaQtlModelFactory: **set(String, String)** - Static method in class org.metaqtl.graph.MetaGraphPar: **setCentroid(double)** - Method in class org.metaqtl.TreeNode: **setChildren(OntologyTerm[])** - Method in class org.thalia.bio.entity.OntologyTerm: Sets the children of the term. **setChromAxe(ChromAxe)** - Method in class org.metaqtl.graph.QtlPartitionLayer: **setChromAxe(ChromAxe)** - Method in class org.metaqtl.graph.QtlTreeLayer: **setChromName(int, String)** - Method in class org.metaqtl.MetaQtlModel: **setCIMiss(int)** - Method in class org.metaqtl.algo.ClustAlgorithm: **setCIMode(int)** - Method in class org.metaqtl.algo.ClustAlgorithm: **setClusterings(List)** - Method in class org.metaqtl.MetaQtlResult: **setClusterings(EMResult[])** - Method in class org.metaqtl.MetaQtlResult: **setCriterion(String)** - Method in class org.metaqtl.MetaQtlModel: Set the criterion name which was used to obtain this model **setCrossDesign(IBioGenome, CrossPopulation)** - Static method in class org.metaqtl.util.GeneticMapProperties: **setCrossType(int)** - Method in class org.thalia.bio.entity.CrossPopulation: **setDF(double)** - Method in interface org.metaqtl.numrec.NumericalUtilities.Function: Sets the derivative of the function at x. **setDubiousMarker(IBioLocus[])** - Method in class org.metaqtl.ChromCluster: This method sets the list of dubious marker. **setDubiousMarker(IBioLocus[])** - Method in class org.metaqtl.MapCluster: This method sets the list of dubious marker. **setEMEps(double)** - Method in class org.metaqtl.algo.QtlClustAlgorithm: **setEMRanStartNumber(int)** - Method in class org.metaqtl.algo.QtlClustAlgorithm: **setFunction(String)** - Method in class org.thalia.bio.entity.Ontology: Set the function of the ontology. **setGeneration(int)** - Method in class org.thalia.bio.entity.Population: **setGenome(IBioGenome)** - Method in class org.thalia.bio.entity.LocusContainer: **setGenome(IBioGenome)** - Method in interface org.thalia.bio.IBioLGroup: **setGroup(IBioLGroup)** - Method in class org.thalia.bio.entity.AlleleContainer: **setGroup(IBioLGroup)** - Method in class org.thalia.bio.entity.Locus: **setGroup(IBioLGroup)** - Method in interface org.thalia.bio.IBioLocus: **setGroups(ArrayList)** - Method in class org.thalia.bio.entity.bean.GeneticMapBean: **setID(long)** - Method in class org.thalia.bio.entity.OntologyTerm: Sets the indentifient of the term. **setIgnore(boolean)** - Method in class org.metaqtl.Qtl: Set the status of the QTL. **setLabelSide(int)** - Method in class org.metaqtl.graph.ChromLayer: **setLabelSide(int)** - Method in class org.metaqtl.graph.ChromShape: **setLoci(IBioLocus[])** - Method in class org.thalia.bio.entity.LocusContainer: **setLoci(IBioLocus[])** - Method in interface org.thalia.bio.IBioLGroup: **setLocus(ArrayList)** - Method in class org.thalia.bio.entity.bean.LGroupBean: **setLocus(IBioLocus)** - Method in interface org.thalia.bio.IBioAllele: **setLogger(Writer)** - Method in class org.metaqtl.algo.MetaAlgorithm: **setLogger(OutputStream)** - Method in class org.metaqtl.algo.MetaAlgorithm: **setLogger(Writer)** - Method in interface org.metaqtl.IMetaAlgorithm: Give a `Writer` to the algorithm in order to write out the log info. **setLogger(OutputStream)** - Method in interface org.metaqtl.IMetaAlgorithm: Give a `OutputStream` to the algorithm in order to write out the log info. **setMappingContext(IBioGenome, int, int)** - Static method in class org.metaqtl.util.GeneticMapProperties: **setMarkerDico(MetaDico)** - Method in class org.metaqtl.algo.MetaMapAlgorithm: **setMarkerDico(MetaDico)** - Method in class org.metaqtl.ChromCluster: **setMarkerDico(MetaDico)** - Method in class org.metaqtl.MapCluster: **setMaxClusterNumber(int)** - Method in class org.metaqtl.algo.QtlClustAlgorithm: **setMetaQtls(Qtl[])** - Method in class org.metaqtl.Chromosome: **setModel(int, int[])** - Method in class org.metaqtl.MetaQtlModel: **setMrkThresh(int)** - Method in class org.metaqtl.ChromCluster: **setMrkThreshold(int)** - Method in class org.metaqtl.algo.MetaMapAlgorithm: **setName(String)** - Method in class org.thalia.bio.entity.bean.BioEntityBean: **setName(String)** - Method in class org.thalia.bio.entity.bean.PropertyBean: **setName(String)** - Method in class org.thalia.bio.entity.BioEntity: **setName(String)** - Method in class org.thalia.bio.entity.BioEntityCollection: **setName(String)** - Method in interface org.thalia.bio.IBioEntity: **setParameter(String)** - Static method in class org.metaqtl.main.MetaMain: **setParentTerm(IBioOntologyTerm)** - Method in class org.thalia.bio.entity.OntologyTerm: Sets the parent of the term. **setPedigree(IBioIndividualTree)** - Method in class org.thalia.bio.entity.Individual: **setPopulation(IBioPopulation)** - Method in class org.thalia.bio.entity.Individual: **setPopulation(IBioPopulation)** - Method in interface org.thalia.bio.IBioIndividual: **setPosition(ILGroupPosition)** - Method in class org.thalia.bio.entity.AlleleContainer: **setPosition(ILGroupPosition)** - Method in class org.thalia.bio.entity.Locus: **setPosition(double)** - Method in class org.thalia.bio.entity.Locus: Set the position of the locus on the linkage group in static mode i.e without uncertainty. **setPosition(double, double)** - Method in class org.thalia.bio.entity.Locus: Set the position of the locus on the linkage group in estimate mode i.e with uncertainty. **setPosition(ILGroupPosition)** - Method in interface org.thalia.bio.IBioLocus: **setPosition(double)** - Method in interface org.thalia.bio.IBioLocus: **setPosition(double, double)** - Method in interface org.thalia.bio.IBioLocus: **setProba(double[][])** - Method in class org.metaqtl.Chromosome: **setProperties(ArrayList)** - Method in class org.thalia.bio.entity.bean.BioEntityBean: **setProperties(Properties)** - Method in class org.thalia.bio.entity.BioEntity: **setProperties(Properties)** - Method in class org.thalia.bio.entity.BioEntityCollection: **setProperties(Properties)** - Method in interface org.thalia.bio.IBioEntity: Sets the properties of the entity. **setPvalue(double)** - Method in class org.metaqtl.algo.QtlProjAlgorithm: **setQtlIdx(int[])** - Method in class org.metaqtl.MetaQtlResult: Set the indices of the Qtl which are implied in this result. **setRatio(double)** - Method in class org.metaqtl.algo.QtlProjAlgorithm: **setRoot(IBioOntologyTerm)** - Method in class org.thalia.bio.entity.Ontology: **setRoot(OntologyTerm)** - Method in class org.thalia.bio.entity.Ontology: **setRoot(IBioOntologyTerm)** - Method in interface org.thalia.bio.IBioOntology: Sets the root of the ontology. **setSD(Double[])** - Method in class org.metaqtl.MetaQtlResult: Set the standard deviations of the Qtl. **setSize(int)** - Method in class org.thalia.bio.entity.Population: Set the population size. **setTerm(String, List)** - Method in class org.metaqtl.MetaDico: **setTraitNames(int, String[])** - Method in class org.metaqtl.MetaQtlModel: **setTree(Tree)** - Method in class org.metaqtl.MetaQtlResult: **setTree(Tree)** - Method in class org.metaqtl.QtlPartition: **setTreeMethod(int)** - Method in class org.metaqtl.algo.QtlTreeAlgorithm: **setValue(String)** - Method in class org.thalia.bio.entity.bean.PropertyBean: **setX(double)** - Method in class org.metaqtl.graph.AnchoredElement: **setX(double)** - Method in class org.metaqtl.graph.ChromAxe: **setX(Double[])** - Method in class org.metaqtl.MetaQtlResult: Set the observed positions of the Qtl. **setX(double)** - Method in class org.metaqtl.TreeNode: **setY(double)** - Method in class org.metaqtl.graph.AnchoredElement: **setY(double)** - Method in class org.metaqtl.TreeNode: **setYMax(double)** - Method in class org.metaqtl.graph.ChromAxe: **setYMin(double)** - Method in class org.metaqtl.graph.ChromAxe: **SF** - Static variable in interface org.thalia.bio.IBioCrossTypes: **shareFlanking** - Variable in class org.metaqtl.Qtl.QTLProj: True if the two maps share a common marker sequence around the QTL. **shortForm()** - Method in class org.metaqtl.main.CmdLineParser.Option: **SIGN(double, double)** - Static method in class org.metaqtl.numrec.NumericalUtilities: Returns abs(a) if b > 0, -abs(a) otherwise. **SINGLE\_COMMON** - Static variable in class org.metaqtl.graph.ChromLayer: **SINGLE\_COMMON\_COLOR** - Static variable in class org.metaqtl.graph.MetaGraphPar: **size** - Variable in class org.metaqtl.Qtl.QTLCross: The number of individuals. **skeleton** - Variable in class org.metaqtl.Chromosome: The skeleton status for this member **sortCluster()** - Method in class org.metaqtl.EMResult: Sort the components in increasing order. **sortLociByPosition(IBioLocus[])** - Static method in class org.thalia.bio.util.BioUtilities: Sort tab with loci **SQR(double)** - Static method in class org.metaqtl.numrec.NumericalUtilities: Returns a^2 **standardError()** - Method in interface org.thalia.bio.util.ILGroupPosition: **standardError()** - Method in class org.thalia.bio.util.LGroupPosition: **STATIC** - Static variable in interface org.thalia.bio.util.ILGroupPosition: **StaticLocusPosition** - Class in org.thalia.bio.util: Class Description Here **StaticLocusPosition(double)** - Constructor for class org.thalia.bio.util.StaticLocusPosition: **stats** - Variable in class org.metaqtl.Qtl: The QTL statistics **stderr** - Variable in class org.thalia.bio.entity.bean.LocusBean: **string2color(String)** - Static method in class org.metaqtl.graph.MetaGraphPar: **string2font(String)** - Static method in class org.metaqtl.graph.MetaGraphPar: **summarize(OutputStream)** - Method in class org.metaqtl.ChromCluster.ClusterGraph: **SVDAlgorithm** - Class in org.metaqtl.algo: This class defines methods to solve usual linear algebra problems by using Singular Value Decomposition (SVD) strategy. **SVDAlgorithm()** - Constructor for class org.metaqtl.algo.SVDAlgorithm: **SVDBackSub(double[][], double[], double[][], double[], int, int)** - Static method in class org.metaqtl.algo.SVDAlgorithm: Solves A.X=B for a vector X, where `A` is specified by the matrix `U`,`V` and the vector `w` as returned by the SVDecomposition() method. **SVDecomposition(double[][], double[][], double[], int, int)** - Static method in class org.metaqtl.algo.SVDAlgorithm: Given a matrix `A`, this routine computes its singular value decomposition A = U.W.V' . **SVDMFit(double[][], double[], double[], int, int)** - Static method in class org.metaqtl.algo.SVDAlgorithm: Given a set of data points y[1..m] with indiviudal weights w[1..m], use chi2 minimization to determine the coefficients a[1..n] of the fitting function y = X\*a. **swap** - Variable in class org.metaqtl.Qtl.QTLProj: True if the flanking marker order are inversed between the skeleton and the original map. **synchChromGraphs(ChromGraph[], Set)** - Static method in class org.metaqtl.main.MetaMain: **SYNONYMS** - Static variable in interface org.thalia.bio.IOntologyTermProperties: The synonyms property for a term in a ontology.

---


|  |  |  |
| --- | --- | --- |
| **Overview** \| Package \| Class \| Use \| **Tree** \| **Deprecated** \| **Index** \| **Help** | |  |
| **PREV LETTER**   **NEXT LETTER** | **FRAMES**    **NO FRAMES**     **All Classes** |  |


A B C D E F G H I K L M N O P Q R S T U V W X Y Z 

---
